# Deep learning-based detection of cerebral microbleeds on 2D T2*-weighted GRE MRI: toward ARIA-H risk assessment in Alzheimer’s treatment

**DOI:** 10.3389/fnagi.2026.1729422

**Published:** 2026-03-23

**Authors:** Soo-Oh Yang, Jehyun Ahn, Young Hee Jung, Hyemin Jang, Duk L. Na, Heejin Kim, Jun Pyo Kim, Sang Won Seo, Kichang Kwak

**Affiliations:** 1 BeauBrain Healthcare, Inc., Seoul, Republic of Korea; 2 Alzheimer’s Disease Convergence Research Center, Samsung Medical Center, Seoul, Republic of Korea; 3 Department of Neurology, Hallym University Sacred Heart Hospital, College of Medicine, Hallym University, Anyang, Republic of Korea; 4 Department of Neurology, Asan Medical Center, Seoul, Republic of Korea; 5 Department of Neurology, Samsung Medical Center, Sungkyunkwan University School of Medicine, Seoul, Republic of Korea; 6 Department of Health Sciences and Technology, SAIHST, Sungkyunkwan University, Seoul, Republic of Korea; 7 Department of Digital Health, SAIHST, Sungkyunkwan University, Seoul, Republic of Korea

**Keywords:** cerebral microbleeds, 2D T2*-weighted GRE MRI, Alzheimer’s disease, ARIA-H, deep learning, YOLO

## Abstract

**Background:**

Amyloid-related imaging abnormalities with hemorrhage (ARIA-H) are a key safety concern in anti-amyloid therapies for Alzheimer’s disease, as they are radiologically indistinguishable from cerebral microbleeds (CMBs). Accurate detection of CMBs is therefore essential for both treatment eligibility assessment and post-treatment safety monitoring. However, manual identification on 2D T2*-weighted gradient-recalled echo (GRE) MRI is labor-intensive and subject to variability.

**Objective:**

To develop and validate an artificial intelligence (AI)-based model for automated CMB detection using only 2D T2*-weighted GRE MRI, which is widely used in clinical settings.

**Methods:**

We implemented a YOLOv11-based deep learning model, preceded by a novel multi-channel preprocessing pipeline that enhances CMB visibility. The model was trained and tested using a dataset of 758 participants, with expert consensus used as the reference standard.

**Results:**

Using the optimized basic preprocessing with super-resolution (BP + SR) pipeline, the model achieved a lesion-level sensitivity of 0.694, precision of 0.705, and F1-score of 0.699. In patient-level analysis for detecting elevated CMB burden (≥4), the system demonstrated sensitivity of 0.933 and specificity of 0.935, supporting reliable stratification of CMB severity. Regional analysis showed sensitivity of 0.625 for lobar CMBs and 0.627 for deep structures.

**Conclusion:**

This study demonstrates the feasibility of robust CMB detection using only 2D T2*-weighted GRE MRI. Based on current performance, we position this system as a decision-support tool for GRE-based CMB screening, in which lesion-level detections may be aggregated to inform patient-level CMB burden relevant to ARIA-H risk stratification, while final ARIA grading and clinical decisions require expert neuroradiological confirmation.

## 1 Introduction

Cerebral microbleeds (CMBs) are small (2–10 mm), chronic hemorrhagic lesions characterized by focal hypointensities on T2*-weighted gradient-recalled echo (GRE) or susceptibility-weighted imaging (SWI) (Agarwal et al., 2024; Charidimou et al., 2018). These lesions reflect hemosiderin-laden macrophage accumulation secondary to blood–brain barrier disruption and are well-established imaging biomarkers of cerebral small vessel disease (Hayden, 2024; Charidimou and Werring, 2011; Shoamanesh et al., 2011). Beyond their vascular relevance, CMBs have been associated with cognitive decline and neurodegenerative conditions, including Alzheimer’s disease (Martinez-Ramirez et al., 2014; van Rooden et al., 2014). Importantly, their topographical distribution—lobar versus deep—can help differentiate between cerebral amyloid angiopathy and hypertensive vasculopathy, adding further clinical value (Charidimou et al., 2015; Poels et al., 2010).

With the advent of disease-modifying therapies (DMTs) targeting *β*-amyloid in Alzheimer’s disease, such as monoclonal antibodies (Salloway et al., 2022; van Dyck et al., 2023), there is growing concern about amyloid-related imaging abnormalities with hemosiderin deposition (ARIA-H), a key safety issue in clinical trials and treatment (Hampel et al., 2020; Sperling et al., 2011). ARIA-H lesions are radiologically indistinguishable from CMBs, complicating safety assessments (Barakos et al., 2013). While recognizing pre-existing CMBs is essential for treatment eligibility and risk stratification (Charidimou et al., 2018), it is even more critical to accurately identify new, treatment-induced ARIA-H events for appropriate therapeutic decision-making and safety monitoring (Cogswell et al., 2022). However, manual detection of CMBs is labor-intensive and prone to inter-rater variability (Gregoire et al., 2009; Cordonnier et al., 2009), especially in real-world clinical practice, highlighting the urgent need for robust, automated systems that can detect CMBs using widely available imaging modalities.

Numerous automated CMB detection systems have been proposed, leveraging deep learning techniques across various imaging sequences (Litjens et al., 2017; Dou et al., 2016; Chen et al., 2019). Most existing methods rely heavily on susceptibility-weighted imaging (SWI), which provides superior contrast for detecting hemorrhagic lesions (Shams et al., 2015; Conijn et al., 2010). However, SWI is not consistently acquired in routine clinical protocols due to its longer scan time and limited availability in certain institutions (Stehling et al., 2008). In contrast, 2D T2*-weighted GRE imaging is more broadly accessible but presents technical challenges for automated detection due to its lower contrast-to-noise ratio and increased incidence of CMB mimics (Nandigam et al., 2009; Goos et al., 2011). Furthermore, many prior models utilize two-stage frameworks—initial candidate detection followed by false positive reduction—introducing complexity in training and potential error propagation between stages (Al-Masni et al., 2020; Liu et al., 2019).

To overcome these limitations, we developed a fully automated CMB detection system that operates solely on 2D T2*-weighted GRE images. Our approach integrates a novel multi-channel preprocessing pipeline tailored to enhance CMB-relevant features and applies a state-of-the-art single-stage detection architecture (YOLOv11) (Khanam and Hussain, 2024) for efficient, end-to-end learning. This design aims to balance sensitivity and specificity without the overhead of multi-stage training, improving clinical scalability. Through rigorous validation on a large institutional dataset, we demonstrate that our GRE-based method achieves competitive performance relative to prior SWI-based approaches, highlighting its potential as a practical tool for widespread clinical use.

## 2 Results

### 2.1 Participant characteristics

A total of 758 participants were included in this study, divided into a training set (*n* = 454), validation set (*n* = 151), and test set (*n* = 153), as summarized in Table 1. The mean age ranged from 73.6 ± 7.0 to 75.0 ± 7.8 years, and the proportion of female participants ranged from 54.9 to 62.0% across the three sets. The number of cerebral microbleeds (CMBs) was highest in the training set (24.2 ± 259.5), followed by the test (14.0 ± 43.3) and validation (8.5 ± 16.9) sets.

**Table 1:** Baseline characteristics and dataset distribution.

Characteristic	CU (*n* = 84)	MCI (*n* = 249)	DAT (*n* = 166)	SVCI (*n* = 206)	Others (*n* = 53)
Demographics					
Age, years	71.7 (7.4)	73.7 (6.9)	74.9 (8.1)	75.1 (6.9)	73.8 (7.1)
Sex, female *n* (%)	52 (61.9)	139 (55.8)	103 (62.0)	136 (66.0)	21 (39.6)
Education, years	10.7	11.2	10.2	8.7	10.5
Handedness, right, *n* (%)	77 (91.7)	237 (95.2)	157 (94.6)	199 (96.6)	47 (88.7)
Clinical measures					
K-MMSE	27.6 (2.6)	25.1 (3.7)	19.1 (5.3)	22.4 (5.0)	20.3 (6.6)
APOE e4 carriers, *n* (%)	22 (26.2)	113 (45.4)	88 (53.0)	58 (28.2)	15 (28.3)
Imaging findings					
CMBs, median (IQR)	1.0 (1.0–3.0)	2.0 (1.0–4.0)	3.0 (1.0–8.8)	6.0 (2.0–18.5)	3.0 (1.0–6.0)
Dataset distribution					
Training set, *n* (%)	52 (61.9)	141 (56.6)	102 (61.4)	128 (62.1)	31 (58.5)
Validation set, *n* (%)	13 (15.5)	57 (22.9)	37 (22.3)	37 (18.0)	7 (13.2)
Test set, *n* (%)	19 (22.6)	51 (20.5)	27 (16.3)	41 (19.9)	15 (28.3)

Data are presented as mean (standard deviation) for continuous variables, *n* (%) for categorical variables, and median (interquartile range) for non-normally distributed continuous variables.

CU, cognitively unimpaired; MCI, mild cognitive impairment; DAT, Alzheimer’s disease dementia; SVCI, subcortical vascular cognitive impairment; K-MMSE, Korean version of mini-mental state examination; APOE, apolipoprotein E; CMB, cerebral microbleed; IQR, interquartile range.

### 2.2 Overall detection performance

The optimized YOLOv11-based model was evaluated on the independent test set of 153 subjects. Using basic preprocessing with super-resolution (BP + SR), the model achieved a sensitivity of 0.694, a precision of 0.705, and an F1-score of 0.699 (Table 2). The average number of false positives per subject (FPavg) was 4.07. Of the 2,138 manually annotated cerebral microbleeds (CMBs), the model correctly detected 1,484 (true positives), missed 654 (false negatives), and generated 622 false positives.

**Table 2:** Performance comparison of different preprocessing strategies on the test dataset.

Preprocessing strategy	TP	FP	FN	Sensitivity	Precision	F1-score	${\mathrm{FP}}_{\mathrm{avg}}$
BP + SR + MIG + YOLOv11	1,491	693	647	0.683	0.697	0.690	4.53
BP+SR+YOLOv11	1,484	622	654	0.694	0.705	0.699	4.07

BP, basic preprocessing; SR, super-resolution; MIG, multi-channel image generation; TP, true positive; FP, false positive; FN, false negative.

To investigate the impact of our proposed multi-channel image generation (MIG) strategy, we conducted an ablation study. When MIG was added (BP + SR + MIG + YOLOv11), sensitivity slightly improved to 0.683 and the number of true positives increased from 1,484 to 1,491. However, this was accompanied by an increase in false positives (from 622 to 693), resulting in reduced precision (0.697) and a lower F1-score (0.690), as summarized in Table 2. These findings suggest that although the multi-channel preprocessing may enhance true lesion contrast, it also increases the likelihood of false positives, particularly for CMB mimics such as vascular structures.

### 2.3 Patient-level performance analysis

To assess clinical applicability at decision-relevant thresholds aligned with Boston Criteria v2.0 and ARIA-H risk assessment, we computed patient-level metrics in the test cohort (*n* = 153). It should be noted that this memory clinic cohort consisted almost entirely of patients with CMBs, as only 1 of 153 subjects presented with zero CMBs. Consequently, specificity and NPV at the ≥1 CMB threshold cannot be reliably estimated from this dataset and require external validation.

However, for detecting elevated CMB burden (≥4 CMBs)—a clinically meaningful threshold for both CAA classification and ARIA-H risk stratification—the analysis provided robust performance estimates comparing patients with low (1–3 CMBs) versus elevated burden (≥4 CMBs). At this threshold, patient-level sensitivity was 0.933, specificity was 0.935, PPV was 0.903, and NPV was 0.956 (Supplementary Table 1).

### 2.4 Regional analysis of detection performance

Regional Analysis of Detection Performance using anatomical brain masks revealed performance variation across CMB locations (Supplementary Table 2). Of note, this analysis includes only lesions that could be anatomically classified within the predefined mask regions; a subset of ground truth CMBs and model detections located at mask boundaries or outside the atlas coverage were excluded, resulting in fewer total lesions compared to the overall detection analysis (Table 2). Regarding lobar CMBs, the model achieved a sensitivity of 0.625 and precision of 0.572. The majority of detected lesions were located in lobar regions, consistent with the high prevalence of CAA in our cohort. In deep structures, the system demonstrated a sensitivity of 0.627 and precision of 0.711, reflecting the more distinctive appearance of hemorrhagic lesions against the deep gray matter background. In contrast, detection performance was lowest for infratentorial CMBs (sensitivity 0.375), which may be attributable to susceptibility artifacts arising from the skull base and surrounding air-bone interfaces (Table 3).

**Table 3:** Performance comparison with existing CMB detection methods.

Type	Reference	Modality	Dataset size (subjects/CMBs)	Resolution (mm^2^)	Sensitivity	Precision	F1-score	${\mathrm{FP}}_{\mathrm{avg}}$
Lesion-level	Myung et al. (2021)	GRE	186/1716	0.63 × 0.63	0.669	0.798	0.728	2.15
	Al-Masni et al. (2020)	SWI + Phase	72/188 (HR)	0.50 × 0.50	0.943	0.619	0.748	1.42
	Suwalska et al. (2022)	SWI	39/144 (DS1)	N/A	0.9	0.220	0.353	0.54
	Kim et al. (2025)	SWI + Phase (3D)	114/365	0.50 × 0.50	0.947	0.780	0.893	0.86
	Our method	GRE	758/8915	0.429 × 0.429–0.469 × 0.469	0.683	0.697	0.690	4.53
Patient-level	Sima et al. (2024) ^*^	GRE	199/NR	NR	0.87^**^	NR	NR	NR

SWI, susceptibility-weighted imaging; GRE, gradient-recalled echo.

Cross-study comparisons are indirect and should be interpreted with caution, as differences in cohort characteristics, imaging protocols, lesion prevalence, and ground truth standards preclude direct equivalence assessment. SWI-based methods benefit from intrinsically higher lesion contrast. ^*^Sima et al. (2024) reported patient-level detection metrics for new ARIA-H (comparing baseline to follow-up MRI), which differs fundamentally from lesion-level CMB detection evaluated in other studies. ^**^ Patient-level sensitivity for detecting any new ARIA-H occurrence. A head-to-head comparison in the same cohort would be required to establish true performance equivalence.

### 2.5 Detection by lesion size

**Figure 1:**
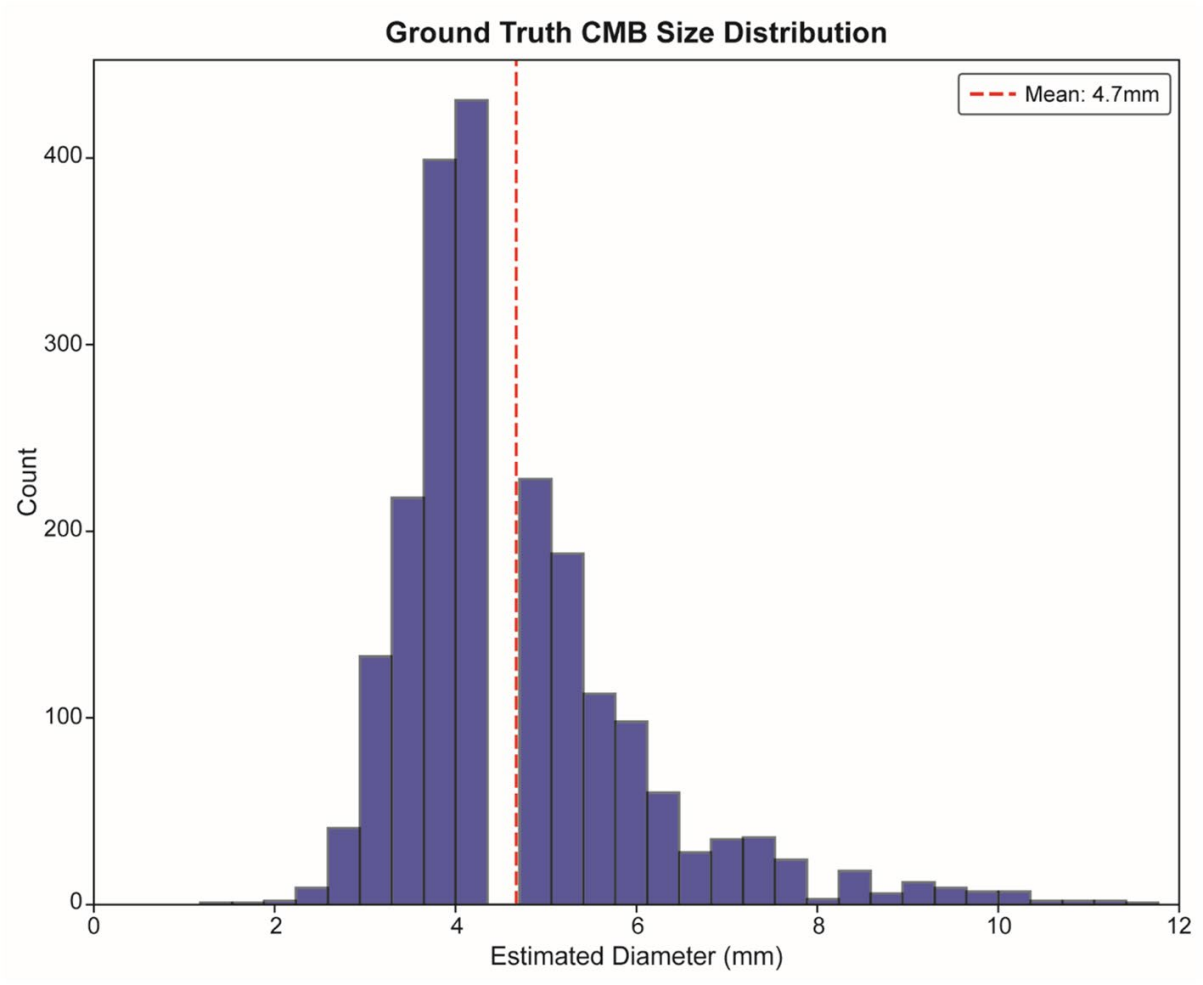
Ground truth CMB size distribution in the dataset. Histogram showing the distribution of CMB diameters across all 8,915 manually annotated lesions in the dataset. The distribution follows a normal pattern with a mean diameter of 4.7 mm (red dashed line), consistent with the established clinical definition of CMBs as 2–10 mm lesions. This size analysis informed the anchor box optimization for our detection model and provided the foundation for size-stratified performance evaluation. CMB, cerebral microbleed.

**Figure 2:**
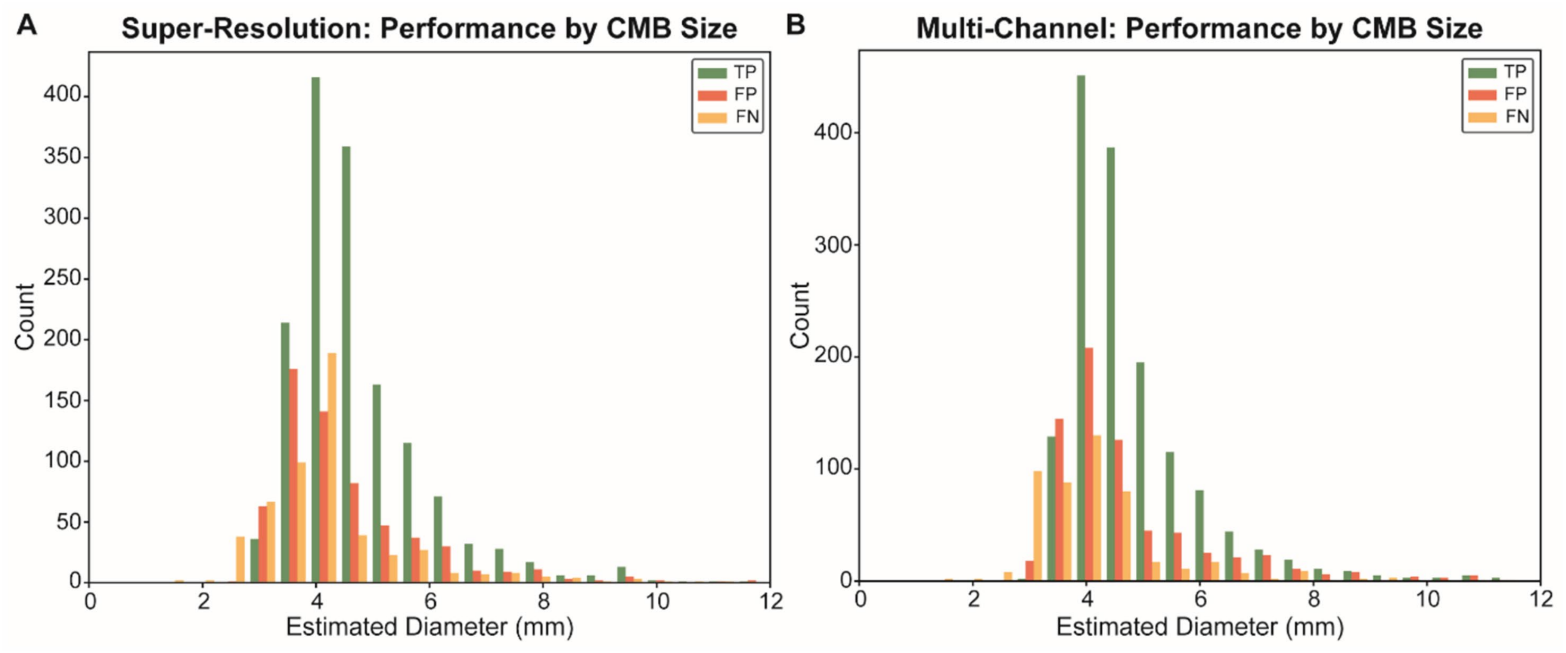
Detection results stratified by CMB size for different preprocessing approaches. Comparison of detection performance across CMB size ranges for super-resolution **(A)** and multi-channel **(B)** preprocessing approaches. Each panel shows the distribution of TP (green), FP (red), and FN (orange) across different estimated diameter ranges. Both approaches demonstrate peak performance in the 4–5 mm range, where most CMBs are concentrated. The multi-channel approach shows increased false positive detections across all size ranges, particularly affecting overall precision. CMBs, cerebral microbleeds; TP, true positive; FP, false positive; FN, false negative.

We further analyzed the detection performance stratified by CMB size to understand model behavior across the lesion spectrum. The size distribution of all 8,915 manually annotated CMBs followed a normal distribution with a mean diameter of 4.7 mm, consistent with the established clinical definition of CMBs (2–10 mm) (Figure 1). This size distribution informed our anchor box optimization and size-stratified evaluation strategy. Detection outcomes across different CMB size bins revealed that both preprocessing strategies showed peak performance in the 4–5 mm range, where most lesions were concentrated (Figure 2). However, the MIG-based approach led to more false positives across all sizes, particularly in the 4–5 mm range.

**Figure 3:**
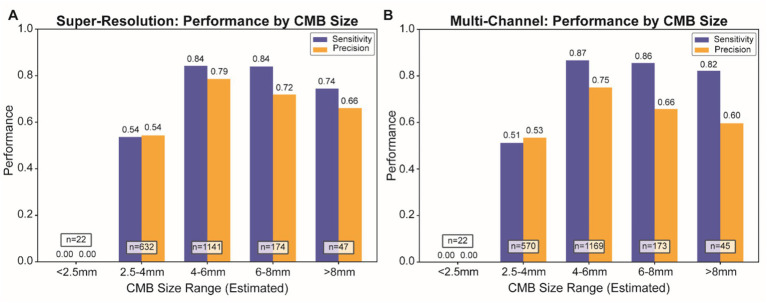
Size-stratified sensitivity and precision metrics for different preprocessing approaches. Performance metrics (sensitivity in blue, precision in orange) across different CMB size categories for super-resolution **(A)** and multi-channel preprocessing **(B)**. Numbers in brackets indicate the sample size for each category. Both methods achieve optimal performance for medium-sized CMBs (4–6 mm), with sensitivity values exceeding 0.84. Performance decreases for very small (<2.5 mm) and very large (>6 mm) lesions, reflecting the challenges associated with detecting lesions at the extremes of the size distribution. CMB, cerebral microbleed.

Sensitivity and precision metrics across different size categories showed that both preprocessing strategies achieved optimal sensitivity (over 0.84) in the 4–6 mm range. Detection of very small (<2.5 mm) and large (>6 mm) lesions was less robust, likely due to their lower frequency and more challenging image characteristics. While sensitivity was comparable, the BP + SR approach demonstrated more stable precision across lesion sizes (Figure 3).

### 2.6 Qualitative assessment

**Figure 4:**
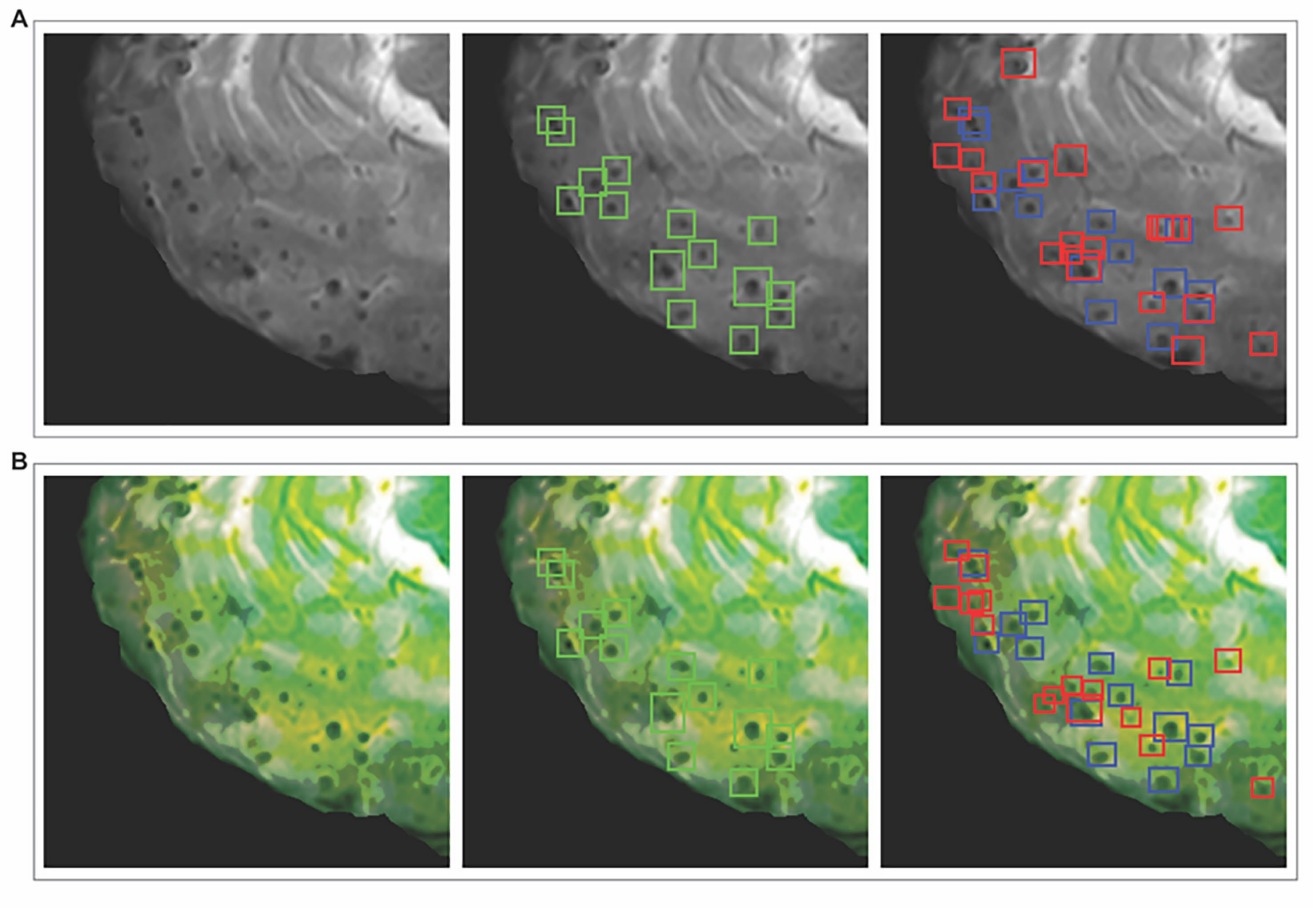
Comprehensive qualitative analysis of CMB detection results representative examples comparing different preprocessing strategies on the same anatomical slice. **(A)** Shows results from the super-resolution preprocessing approach: (left) original 2D T2*-weighted GRE with super-resolution enhancement, (middle) ground truth annotations, and (right) model detection results. **(B)** Displays results from the multi-channel preprocessing approach: (left) preprocessed image after multi-channel enhancement, (middle) ground truth annotations, and (right) corresponding detection results. Green bounding boxes indicate ground truth annotations established by expert consensus, blue boxes show true positive detections (correctly identified CMBs), and red boxes represent false positive detections (incorrectly identified structures). The comparison illustrates how the multi-channel approach enhances CMB visibility but also amplifies mimicking structures, leading to increased false positive detections compared to the super-resolution approach. CMB, cerebral microbleed; GRE, gradient-recalled echo.

Visual inspection of representative detection results further highlighted the contrasting characteristics of the two preprocessing approaches. The BP + SR approach provided more conservative detections, with fewer false positives and better correspondence with expert annotations. In contrast, the BP + SR + MIG approach enhanced lesion conspicuity, but also highlighted non-lesional structures, resulting in higher false positive rates despite slight gains in sensitivity (Figure 4).

## 3 Discussion

Recently, FDA-cleared commercial solutions such as Icobrain ARIA (icometrix) have emerged for ARIA-related imaging assessment (Sima et al., 2024). While these solutions offer robust performance, they typically require specific protocols. Similarly, commercial AI solutions developed in Korea (e.g., Neurophet) have been introduced for automated CMB detection. However, detailed technical specifications and peer-reviewed validation data for these systems are not publicly available, precluding a direct methodological comparison. Based on available information, such systems often leverage susceptibility-weighted imaging (SWI) or multi-modal inputs for enhanced sensitivity. In contrast, the present study adopts a GRE-only approach that addresses a complementary clinical need by enabling automated CMB screening in settings where only standard 2D T2*-weighted GRE is available and SWI acquisition is not routinely performed. This positioning reflects our focus on real-world accessibility and the development of a decision-support framework to aid ARIA-H relevant safety monitoring, rather than a direct replacement of existing commercial systems. This study successfully developed and validated an automated cerebral microbleed (CMB) detection system using 2D gradient-echo T2*-weighted MRI in a large, clinically relevant cohort of 758 participants. Our model demonstrated robust performance (F1-score 0.699; sensitivity 0.694), even without using SWI or 3D acquisitions. By focusing on 2D T2*-weighted GRE, a sequence broadly available in routine practice, our model is readily applicable to real-world clinical settings. This accessibility is particularly valuable in the context of Alzheimer’s disease (AD) treatment, where monitoring for amyloid-related imaging abnormalities with hemorrhage (ARIA-H) is essential for patient safety.

ARIA-H lesions, which appear radiologically indistinguishable from CMBs, have emerged as a major safety concern with the advent of anti-amyloid monoclonal antibody therapies (Hampel et al., 2023). While baseline detection of pre-existing CMBs is important for determining treatment eligibility and stratifying hemorrhagic risk, longitudinal monitoring of new CMB-like lesions is even more critical for identifying potential ARIA-H events during treatment (Sperling et al., 2011). Automated CMB detection from 2D T2*-weighted GRE —a sequence commonly available in AD clinical trials and memory clinic protocols—can therefore facilitate early ARIA-H detection, supporting more informed therapeutic decision-making and enhanced safety surveillance (Tsuchida et al., 2024). In practice, patient-level CMB burden can be derived by aggregating lesion-level detections above a confidence threshold. For ARIA-H severity assessment, lobar CMB counts would then be compared against established clinical cutoffs corresponding to mild (1–5 new microhemorrhages), moderate (6–10), and severe (>10 or macrohemorrhage) categories. However, the current FPavg of approximately 4 indicates that fully automated ARIA-H grading remains limited, particularly for patients near decision-relevant thresholds (e.g., distinguishing 3 versus 5 lobar CMBs), where individual false positives could alter risk categorization. Therefore, pending external validation and formal patient-level safety evaluation, we position this system as an initial screening tool to flag patients warranting detailed expert review, rather than as an autonomous classifier for ARIA-H severity.

Our investigation into a novel multi-channel preprocessing pipeline yielded meaningful insights. Ablation analysis showed this method enhanced subtle CMB visibility and improved true positive rates. However, it also increased false positives, likely due to amplified signals from CMB mimics. This finding highlights the need for further optimization—particularly of Z-score thresholding, morphological filters, and intensity scaling. The promising results suggest that a more data-driven or learnable preprocessing approach could enhance performance without compromising precision.

In comparing our model with prior work, we note that despite relying on single-stage detection and lower-contrast GRE input, our system demonstrated competitive performance relative to other GRE- and SWI-based models. Notably, our precision was higher than in many SWI-dependent pipelines, which often rely on complex two-stage detection or image fusion. Our simplified yet effective pipeline could thus promote broader clinical implementation without increasing computational burden. The modestly elevated false positive rate may partially reflect the use of 2D acquisition, which lacks the contextual depth of 3D imaging.

Size-stratified performance analysis revealed that both true and false positives were concentrated in the 4–5 mm range. Model performance was optimal for medium-sized CMBs (4–6 mm), while smaller (<2.5 mm) and larger (>6 mm) lesions posed greater detection challenges. These results are consistent with the expected CMB size distribution in aging and cerebrovascular populations. Future work could explore size-aware training strategies or ensemble models to improve detection across a broader size spectrum.

Although the multi-channel pipeline offers clear benefits in enhancing sensitivity, its amplification of mimics points to an urgent need for more refined false positive reduction strategies. Integrating spatial context—via hybrid 2D–3D architectures—or post-processing modules to filter likely mimics may yield further improvements. Embedding learnable preprocessing within the detection network itself is also a promising direction.

This study is strengthened by its use of a large, clinically relevant dataset and a single-stage GRE-based detection pipeline tailored to real-world imaging protocols. However, several limitations warrant consideration. First, although the dataset size is substantial, all imaging data were acquired from a single 3 T Philips scanner, and no external or multi-scanner validation was performed. The preprocessing pipeline—including intensity normalization and super-resolution—was designed to standardize image characteristics and may partially mitigate scanner-related variations by reducing contrast and resolution differences across acquisitions. However, whether these steps sufficiently address domain shift across different vendors (e.g., GE, Siemens), field strengths (1.5 T vs. 3 T), or acquisition parameters remains empirically untested. Future multi-center validation incorporating diverse imaging protocols will be essential to establish generalizability and to determine whether additional domain adaptation strategies are required. Second, this study evaluates cross-sectional CMB detection from single time-point. We did not assess longitudinal identification of newly emergent, treatment-related ARIA-H, which requires temporal analysis across serial imaging during anti-amyloid therapy. Therefore, the present results primarily reflect baseline CMB detection performance, while direct evaluation of ARIA-H incidence or progression would require longitudinal analysis. Third, the average number of false positives per subject (FPavg = 4.07), while acceptable, suggests that distinguishing true CMBs from mimics remains a challenge on 2D T2*-weighted GRE images. Although individual false-positive detections occur, patient-level analysis (Supplementary Table 1) demonstrates that at the clinically relevant threshold of ≥4 CMBs, the system achieves high specificity (0.935). This suggests that while individual false positives occur, they do not substantially misclassify patients into high-risk categories, supporting the system’s utility for CMB-based risk stratification. Nevertheless, further reduction of false positives, potentially through advanced post-processing or hybrid 2D–3D architectures remains an important direction for future work.

## 4 Conclusion

This study demonstrated that an automated CMB detection system using only 2D T2*-weighted GRE MRI, coupled with a YOLOv11-based detector and optimized preprocessing, can achieve reliable performance comparable to prior approaches. By facilitating the detection of CMBs—including those resembling ARIA-H—this GRE-based, single-stage pipeline provides a clinically feasible solution for routine screening and safety monitoring, especially in patients undergoing anti-amyloid therapy. While our approach improves sensitivity, further refinement is needed to reduce false positives and support broader clinical adoption.

## 5 Materials and methods

### 5.1 Participants

Participants were recruited from the Memory Clinic of Samsung Medical Center between September 2007 and November 2022 as part of an ongoing clinical research program. A total of 758 individuals who underwent 3 T brain MRI, including gradient-recalled echo (GRE) T2*-weighted sequences, were included in this study. The cohort consisted of 84 cognitively unimpaired (CU) individuals, 249 patients with mild cognitive impairment (MCI), 166 patients diagnosed with Alzheimer’s disease dementia (DAT), 206 patients with subcortical vascular cognitive impairment (SVCI), and 53 patients with other types of dementias. CU was defined as the absence of objective cognitive impairment in any domain on a comprehensive neuropsychological battery, with performance above −1.0 standard deviations (SD) in memory and above −1.5 SD in other domains based on age- and education-adjusted norms (McKhann et al., 2011). MCI was diagnosed in individuals with objective impairment in one or more cognitive domains, while functional independence in daily activities was preserved. DAT was diagnosed based on the 2011 National Institute on Aging and Alzheimer’s Association (NIA-AA) criteria (McKhann et al., 2011), characterized by progressive memory-dominant cognitive decline and impairment in daily functioning. SVCI, encompassing both subcortical vascular MCI and subcortical vascular dementia, was diagnosed in individuals with (1) subjective cognitive complaints, (2) objective cognitive impairment below the 16th percentile in any domain, and (3) severe ischemia on brain MRI, defined as periventricular white matter hyperintensities >10 mm and deep white matter hyperintensities >25 mm, based on modified Fazekas criteria (Fazekas et al., 1993). All diagnoses were established via multidisciplinary consensus conferences, based on clinical interviews, neurological examinations, and neuropsychological test results. Participants with major structural brain lesions that could interfere with microbleed detection (e.g., large infarctions, brain tumors, or extensive hemorrhages), or with poor image quality due to motion artifacts or technical problems, were excluded from analysis.

The study protocol was approved by the Institutional Review Board of Samsung Medical Center. Written informed consent was obtained from all participants in accordance with the Declaration of Helsinki.

### 5.2 Image acquisitions

All participants underwent brain MRI scans using a 3.0 Tesla Philips scanner (Philips Medical Systems, Best, The Netherlands). The imaging protocol consisted of 2D T2*-weighted GRE sequences for cerebral microbleed detection. The 2D T2*-weighted GRE images were acquired in the axial plane with a slice thickness of 5.0 mm and spacing between slices of 6.5 mm, repetition time (TR) ranging from 654.4 to 662.0 ms, echo time (TE) of 16.1 ms, flip angle of 18°, and acquisition matrix of 256 × 249 to 320 × 256 (reconstructed to 512 × 512 or 560 × 560) over a field of view of 240 mm. The sequences utilized a 2D gradient-recalled acquisition with spoiled gradient-echo technique and spectral presaturation for fat suppression. This imaging protocol was standardized across all participants and has been previously validated in studies evaluating hemorrhagic markers in cerebral amyloid angiopathy.

### 5.3 CMB rating and annotation

All 2D T2*-weighted GRE images were visually inspected to ensure adequate image quality for cerebral microbleed (CMB) analysis. CMBs were defined according to established consensus criteria as small (2–10 mm), round or ovoid, hypointense lesions with blooming artifacts on 2D T2*-weighted GRE images, located in the brain parenchyma, and distinct from vascular flow voids, calcifications, iron deposits, or other imaging artifacts. Lesions were included only if they were at least half surrounded by brain tissue and showed homogeneous signal loss. Ground truth annotations were established through independent review and subsequent consensus by three experienced neurologists (J. A., H. K., and J. P. K.) who were blinded to clinical information. Manual segmentation was performed for each CMB, and bounding boxes were generated from the segmentation masks. For model training, these annotations were converted to the YOLO format, with bounding box coordinates normalized relative to image dimensions to ensure consistency across varying matrix sizes. A total of 2,138 CMBs were identified across all participants and included in the final dataset for model development and evaluation. The inter-rater reliability for lobar CMBs was high, with intra-class correlation coefficients (ICCs) ranging from 0.87 to 0.91. It should be noted that GRE-based annotation may undercount CMBs relative to SWI-augmented reads, as SWI provides superior sensitivity for small or subtle lesions. Thus, our ground truth represents lesions detectable on GRE imaging, which is the clinically relevant reference for evaluating GRE-based detection systems.

### 5.4 Image preprocessing

The initial preprocessing stage aimed to standardize the input data. First, non-brain tissue was removed using FSL’s Brain Extraction Tool (BET) (Smith, 2002) with a fractional intensity threshold of 0.5. Subsequently, image intensities were normalized using a percentile-based scaling method (1st and 99th percentiles) to ensure robustness against outliers. To address the challenge of detecting small CMBs in images with relatively thick slices, we integrated a super-resolution step using the Enhanced Deep Residual Networks (EDSR) (Lim et al., 2017) 2x model available in OpenCV. This process enhances spatial resolution, which makes the subtle features of small CMBs more discernible to the detection network. Finally, all volumes were resampled to a uniform voxel resolution of 0.429 × 0.429 × 6.5 mm^3^ using trilinear interpolation to standardize spatial dimensions across the dataset.

### 5.5 Model framework

**Figure 5:**
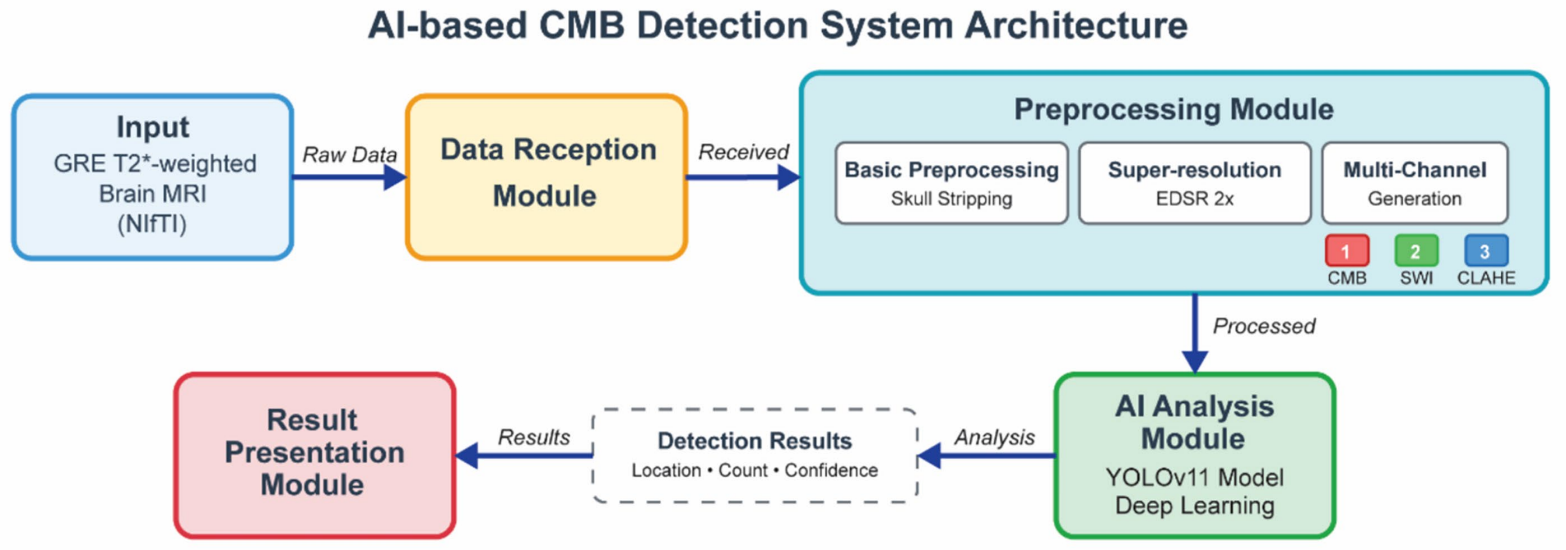
Overview of the proposed artificial intelligence-based automated CMB detection system architecture. The system architecture consists of four integrated modules working in sequence. The data reception module accepts 2D T2*-weighted GRE brain MRI scans in NIfTI format. The preprocessing module implements a multi-step pipeline including basic normalization, super-resolution enhancement, and optional multi-channel image generation. The AI analysis module employs a YOLOv11-based deep learning model to identify CMB locations and assign confidence scores to detected lesions. The result presentation module aggregates and visualizes the analysis outputs, displaying CMB locations, counts, and confidence scores. The workflow demonstrates the complete pipeline from raw MRI input to final detection results. MRI, magnetic resonance imaging; AI, artificial intelligence; CMB, cerebral microbleed; GRE, gradient-recalled echo; EDSR, enhanced deep super-resolution network; SWI, susceptibility-weighted imaging; CLAHE, contrast limited adaptive histogram equalization.

This study was designed to develop and validate an AI-based model for the automated detection of CMBs from clinical brain MRI data. The model architecture, illustrated in Figure 5, consists of four integrated modules that work in sequence. It begins with the Data Reception Module, which accepts 2D T2*-weighted GRE brain MRI scans in the standard NIfTI format. These inputs are then passed to the Preprocessing Module, where a multi-step pipeline is applied, including basic normalization, super-resolution, and our novel multi-channel image generation. The processed images are subsequently analyzed by the AI Analysis Module, which leverages a YOLOv11-based deep learning model to detect candidate CMBs, estimate their locations, and assigning confidence scores. Finally, the Result Presentation Module aggregates the detection outputs and visualizes the results, clearly indicating the location and count of detected CMBs. The overall workflow proceeds from data reception to preprocessing, followed by AI-driven analysis.

### 5.6 Multi-channel image generation

A key novelty of our work is the multi-channel image generation (MIG) technique, which performs a three-channel preprocessing step to create a rich, composite representation from the original 2D T2*-weighted GRE images. Each channel emphasizes different features relevant to CMB detection: Channel 1 (CMB Selective Enhancement): This channel isolates and enhances hypointense regions characteristic of CMBs. It is generated through a series of steps involving Z-score normalization, thresholding (Z-score < −1.0), morphological filtering to retain objects within the typical CMB size range (1–10 mm), and a final distance transformation to encode spatial information. Channel 2 (SWI-like Contrast Enhancement): This channel aims to mimic the high-contrast appearance of CMBs on SWI. It applies a square-root intensity transformation followed by selective attenuation of pixels below the 30th intensity percentile (P_30) to amplify susceptibility effects. A final Gaussian smoothing (*σ* = 0.5) reduces noise. Channel 3 (CLAHE Enhanced Original): To preserve fine anatomical details that might be lost in the other channels, this channel applies Contrast Limited Adaptive Histogram Equalization (CLAHE) (Zuiderveld, 1994) to the normalized input image, enhancing local contrast.

### 5.7 Deep learning model architecture

Our detection model is adapted from the YOLO family of architectures, specifically using a YOLOv11 implementation known for its balance of speed and accuracy. We configured the model to accept a 1,024 × 1,024 pixel, 3-channel input corresponding to our preprocessed images. The network’s anchor boxes were adapted to better match the typical size distribution of CMBs. To handle the profound class imbalance between CMBs and background, we employed a composite loss function. This function combines focal loss for the classification task, which down-weights the loss from easily classified background examples, and Complete IoU (CIoU) loss for the bounding box regression. The CIoU loss offers a more comprehensive regression metric by accounting for the distance, overlap, and aspect ratio consistency between predicted and ground truth boxes. The total loss was a weighted sum of the classification, regression, and objectness loss components.

### 5.8 Implementation

The model implementation was developed using the Ultralytics YOLOv11 framework with PyTorch 2.4.0 and torchvision 0.19.0. All experiments were conducted on a Linux environment (Ubuntu 22.04.5 LTS) equipped with a single NVIDIA L40S GPU (46GB VRAM). The model was trained for 200 epochs using the AdamW optimizer (Loshchilov and Hutter, 2017) with a cosine annealing learning rate scheduler and a batch size of 8. To enhance model generalization, we applied a suite of data augmentation techniques, including random rotations, brightness and contrast adjustments, horizontal flipping, and the addition of Gaussian noise. Early stopping with a patience of 50 epochs was used to prevent overfitting. The initial learning rate was set to 0.001, with weight decay of 5 × 10^−4^ to prevent overfitting. Momentum was set to 0.937 for enhanced convergence stability. The confidence threshold for inference was set to 0.2, with an IoU threshold of 0.5 for non-maximum suppression. Bounding box scale factors were set to 2.0 to accommodate CMB size variations. Image preprocessing utilized OpenCV 4.11.0.86 for multi-channel generation and normalization operations. All training utilized automatic mixed precision (AMP) for memory optimization and computational efficiency.

### 5.9 Evaluation metrics

Model performance was evaluated using standard object detection metrics computed at both object-level and patient-level granularities. Primary metrics include: — These standard metrics are defined in Equations (1–4):


$$\text{Sensitivity}\;(\text{Recall})=\frac{\mathrm{TP}}{\mathrm{TP}+\mathrm{FN}}$$ (1)

$$\text{Precision}\;(\mathrm{PPV})=\frac{\mathrm{TP}}{\mathrm{TP}+\mathrm{FP}}$$ (2)

$$F1-\text{score}=\frac{2\times \text{Precision}\times \text{Recall}}{\text{Precision}+\text{Recall}}$$ (3)

$${\mathrm{FP}}_{\mathrm{avg}}=\frac{\mathrm{FP}}{\text{Number of subjects}}$$ (4)

where TP, TN, FP, and FN represent True Positives, True Negatives, False Positives, and False Negatives, respectively. A detection was considered a true positive when the predicted bounding box achieved an Intersection over Union (IoU) with the ground truth bounding box exceeding 0.5.

## Data Availability

The data analyzed in this study is subject to the following licenses/restrictions: the anonymized data for the analyses presented in this report are available from the corresponding author on reasonable request. Requests to access these datasets should be directed to kichang.kwak@beaubrain.bio.
