# Supplementary material for: Deep learning-based detection of cerebral microbleeds on 2D T2*-weighted GRE MRI: toward ARIA-H risk assessment in Alzheimer’s treatment
Source: Front Aging Neurosci. 2026 Mar 23;18:1729422. doi: 10.3389/fnagi.2026.1729422 (PMC13050780; doi:10.3389/fnagi.2026.1729422)
Supplement: Supplementary file 1 [file Data_sheet_1.docx]

**Supplementary Table 1.** Patient-Level Performance

| **Threshold** | **Sensitivity** | **Specificity** | **PPV** | **NPV** | **TP/TN/FP/FN** |
| --- | --- | --- | --- | --- | --- |
| ≥1 CMB | 0.941 | N/A* | N/A* | N/A* | 143/1/0/9 |
| ≥4 CMBs | 0.933 | 0.935 | 0.903 | 0.956 | 56/87/6/4 |

Performance metrics are reported for thresholds of ≥1 and ≥4 CMBs. Specificity at the ≥1 threshold is not reported due to the absence of CMB-negative controls in the cohort. The ≥4 CMB threshold corresponds to a clinically relevant cutoff commonly used for ARIA-H risk stratification in anti-amyloid therapy. Abbreviation: CMB=cerebral microbleeds, PPV=positive predictive value, NPV=negative predictive value, TP=true positive, FP=false positive, FN=false negative.

**Supplementary Table 2.** Regional Detection Performance

| **Region** | **Sensitivity** | **Precision** | **TP** | **FP** | **FN** |
| --- | --- | --- | --- | --- | --- |
| Lobar | 0.625 | 0.572 | 1,043 | 779 | 627 |
| Deep | 0.627 | 0.711 | 64 | 26 | 38 |
| Infratentorial | 0.375 | 0.231 | 3 | 10 | 5 |
| Total* | 0.587 | 0.577 | 1,111 | 816 | 782 |

*Regional analysis includes only lesions that could be anatomically classified within the brain mask regions. A subset of ground truth CMBs (n=373) and model detections located outside the predefined anatomical masks (e.g., at mask boundaries or in regions not covered by the atlas) were excluded from this analysis. Overall detection performance including all lesions is reported in Table 2.

Abbreviation: TP=true positive, FP=false positive, FN=false negative
